# DNA Electrophoretic Migration Patterns Change after Exposure of Jurkat Cells to a Single Intense Nanosecond Electric Pulse

**DOI:** 10.1371/journal.pone.0028419

**Published:** 2011-12-02

**Authors:** Stefania Romeo, Luigi Zeni, Maurizio Sarti, Anna Sannino, Maria Rosaria Scarfì, P. Thomas Vernier, Olga Zeni

**Affiliations:** 1 CNR – Institute for Electromagnetic Sensing of Environment, Naples, Italy; 2 Department of Information Engineering, Second University of Naples, Aversa, Italy; 3 Department of Pharmaceutical Science, University of Salerno, Fisciano, Italy; 4 Ming Hsieh Department of Electrical Engineering, Viterbi School of Engineering, University of Southern California, Los Angeles, California, United States of America; University of California at Berkeley, United States of America

## Abstract

Intense nanosecond pulsed electric fields (nsPEFs) interact with cellular membranes and intracellular structures. Investigating how cells respond to nanosecond pulses is essential for a) development of biomedical applications of nsPEFs, including cancer therapy, and b) better understanding of the mechanisms underlying such bioelectrical effects. In this work, we explored relatively mild exposure conditions to provide insight into weak, reversible effects, laying a foundation for a better understanding of the interaction mechanisms and kinetics underlying nsPEF bio-effects. In particular, we report changes in the nucleus of Jurkat cells (human lymphoblastoid T cells) exposed to single pulses of 60 ns duration and 1.0, 1.5 and 2.5 MV/m amplitudes, which do not affect cell growth and viability. A dose-dependent reduction in alkaline comet-assayed DNA migration is observed immediately after nsPEF exposure, accompanied by permeabilization of the plasma membrane (YO-PRO-1 uptake). Comet assay profiles return to normal within 60 minutes after pulse delivery at the highest pulse amplitude tested, indicating that our exposure protocol affects the nucleus, modifying DNA electrophoretic migration patterns.

## Introduction

Intense nanosecond pulsed electric fields (nsPEFs) trigger multiple responses in mammalian cells [1], including primary effects on the plasma membrane [2]–[5], sub-cellular membranes and organelles [6]–[9] and secondary effects such as nuclear granulation, calcium release, and apoptosis [6], [10], [11]. Although the mechanisms and dynamics of nsPEF-induced perturbations on intracellular processes are not fully understood, they are under active investigation for applications in cancer therapy, genetic engineering, and cell biology.

The extreme variability of the exposure conditions (pulse amplitude, duration, number, and repetition rate) reported in the literature makes direct comparisons among the biological effects described by different groups difficult. Recently, Ibey and co-workers have proposed absorbed dose (AD), which takes into account both the electric field and the pulse duration, as a reliable metric for a more systematic characterization and comparison of bioeffects observed under different exposure conditions [5]. However, although a widely accepted set of classification and characterization criteria for bioelectric effects has not been established, two dominant lines of investigation can be identified in the literature on nsPEFs: the first addresses the development of biological and clinical applications of nanoelectropulses; the second is aimed at investigating the underlying mechanisms leading to nsPEF-induced bio-effects.

A number of authors have reported the biological effects of nsPEFs in very heavy exposure conditions, in terms of pulse count (tens to hundreds), amplitude (up to 15 MV/m), and duration (up to 300 ns). These efforts are directed at the application of nsPEFs in cancer therapy, as a non-pharmacological, non-thermal alternative treatment that targets cancer hallmarks like apoptosis evasion and sustained angiogenesis [12]–[14].

Milder exposures (fewer pulses, lower amplitudes, shorter durations) have been explored by several groups aiming to understand the interactions between the electric field and cellular structures. Recent studies have shown that even very short nsPEFs can significantly affect the plasma membrane, inducing permeabilization not detectable with conventional methods, such as propidium iodide (PI) uptake. Pakhomov and co-workers have demonstrated that a single 60 ns, 1.2 MV/m pulse can cause a profound and long-lasting (minutes) reduction of the cell membrane electrical resistance, accompanied by the loss of the transmembrane electrical potential, both detected by means of the patch clamp technique [15]. The formation and lifetime of nanopores under these conditions was characterized by monitoring thallium (Tl^+^) influx as an indicator of nanoporation under conditions where no PI uptake is observed [16], [17]. An increase in intracellular Ca^2+^ concentration, assessed by Calcium Green fluorescence, was also reported by Vernier and co-workers in Jurkat cells exposed to 10, 30 ns, 2.5 MV/m pulses [10] and in bovine adrenal chromaffin cells exposed to a single 4 ns, 8 MV/m pulse [18]. The same authors also detected phosphatidylserine externalization in cells exposed to pulsed fields from 3 to 30 ns and 2 to 8 MV/m, observing that shorter pulses require higher fields, consistent with a time-dependent charging of the membrane dielectric. This membrane reorganization was accompanied by the influx of the fluorochrome YO-PRO-1 after exposure of Jurkat cells to 30, 4 ns pulses at fields above 6 MV/m and high pulse repetition rates (1 kHz), with higher uptake at higher repetition rates, and again in the absence of significant PI uptake [3]. Electric pulses as short as 3 ns, with adequate pulse amplitude or number, have been shown to induce effects similar to those observed with longer duration, as long as more sensitive detection systems are available [4].

In this work, relatively mild exposure conditions have been investigated to provide insight into weak, reversible effects, laying a foundation for a better understanding of the interaction mechanisms and kinetics underlying nsPEF bio-effects. This knowledge is essential for the effective application of nsPEF technology in the laboratory and in the clinic. For this purpose, we exposed Jurkat cells (human lymphoblastoid T cells) to single pulses of 60 ns duration and 1.0, 1.5 and 2.5 MV/m amplitude and observed the effects on plasma membrane permeability and nuclear DNA electrophoretic mobility over time after the exposure.

## Materials and Methods

### 1. Cell line and culture conditions

Jurkat cells, kindly provided by Dr. I. Tedesco (CNR-Institute of Food Science and Technology, Avellino, Italy), were seeded in complete medium composed by RPMI 1640, supplemented with 10% heat-inactivated phoetal bovine serum (FBS), both from BioWhittaker (Verviers, Belgium), 1% L-glutamine, 0.5% penicillin-streptomycin, both from Gibco (Milan, Italy), at a density of 2−3×10^5^ cells/mL, and maintained in exponential growth phase at 37°C in a humidified 5% carbon dioxide atmosphere [19]. Before experiments, cell viability was verified to be higher than 85% by means of the trypan blue (BDH, Poole, England) exclusion method.

### 2. Exposure system and procedure

The electric pulse exposure system consisted of a coaxial cable-based Blumlein pulse-forming network matched to the biological load in electroporation cuvette. A Blumlein pulse forming network is usually configured with a high-voltage source charging paired transmission lines, series-connected to the load, which has an impedance equal to twice the characteristic impedance of each transmission line. One side of the Blumlein is connected to a switch, which is open during the charging phase, and then closed to deliver the pulse to the load. This configuration provides rectangular pulses with an amplitude equal to the charging voltage and a width fixed by the propagation delay of the transmission line [20]. Commercially available electroporation cuvettes (Aurora Borealis, NL) having electrode gap of 1 mm were employed.

For the present work, two sets of 50 Ω RG213 coaxial cables connected in parallel were employed to set up two 12.5 Ω transmission lines matched to the 25 Ω biological load. The matching between the transmission lines and the load was achieved by following the standard formula for calculating the load impedance, R_L_ = ρ (d/A), where ρ (100 Ω·cm) is the resistivity of the cell suspension buffer, *d* (0.1 cm) is the gap between the electrodes, and *A* (0.4 cm^2^) is the electrode area covered with the liquid.

A cable length of 12 m was chosen in order to generate 60 ns pulses (the input to output delay of the cable is 5 ns/m), with an amplitude of up to 2.5 kV (maximum electric field 2.5 MV/m across the electrode gap).

For experiments, after calibration of the equipment with PBS (BioWhittaker), 40 µL aliquots of cell suspension in PBS (8×10^6^/mL) in 0.1 cm electroporation cuvettes were exposed and sham-exposed to nsPEFs.

### 3. Cell viability and growth

The impermeability of healthy cells to trypan blue dye was employed to assess cell viability and growth. After nsPEF exposure, cells were seeded in complete culture medium at a density of 3×10^5^ cells/mL. After 24 h and 48 h of growth, cell aliquots were collected and treated with 0.4% (w/v) trypan blue just prior to counting with a Burker hemocytometer under a light microscope (Leica Microsystems, Wetzlar, Germany). The number of viable cells was recorded for each time point to assess cell growth. Cell viability was calculated as the fraction of viable cells in the total population, expressed as percentage.

### 4. Plasma membrane integrity

Propidium iodide (PI, Sigma, St. Louis, MO) and YO-PRO-1 (Invitrogen, Eugene, OR, USA) were used as indicators of plasma membrane integrity. PI is membrane-impermeant and generally excluded from viable cells, but it passes through permeabilized membranes and fluoresces on binding to nucleic acids. YO-PRO-1, a more sensitive detector of membrane permeabilization, is a normally cell-impermeant, monomeric, cyanine dye with a strong binding affinity to nucleic acids [4], [21]. Both dyes can be excited by the 488 nm line of an argon ion laser and are suitable for flow cytometry.

The direct effect of nsPEF on plasma membrane integrity was determined by pulsing cells in presence of 1.5 µM PI or 1 µM YO-PRO-1. To monitor plasma membrane permeabilization, after exposure, cells were re-suspended in complete medium and placed in CO_2_ incubator, then each dye was added at specified times post pulse. Cell suspensions were analyzed with a flow cytometer (FACScalibur, Becton & Dickinson, San Jose, CA). 10,000 events were acquired using FL1 and FL2 channels for YO-PRO-1 and PI, respectively, and analyzed by Cell Quest Pro software.

### 5. DNA migration pattern

The alkaline comet assay was used to evaluate effects of nsPEFs on cellular DNA fragmentation, cross-linking, and aggregation. The assay, developed by Singh and co-workers [22] and previously described in detail [23] was implemented as follows. After exposure, cells to be immediately processed were directly spun down, while cells to be processed over time were spun down after re-suspension in complete medium in CO_2_ incubator for the requested time. For the assay, cells were re-suspended in low melting point agarose (LMA; 0.7% w/v; 37°C) and sandwiched between a lower layer of normal melting agarose (NMA; 1% w/v) and an upper layer of LMA on microscope slides. Agarose (both normal and low melting point) was from Bio-Rad laboratories (GmbH, Munich, Germany). Following overnight immersion in cold lysing solution, made up by 2.5 M NaCl (Carlo Erba, Milan, Italy), 100 mM Na_2_EDTA, 25 mM NaOH (both from Baker, Deventer, The Netherlands), 10 mM Tris pH 10 (BDH), with 1% Triton X-100 (Sigma) and 10% dimethyl sulfoxide (DMSO, Baker), DNA was unwound for 60 min at 4°C in alkaline electrophoresis buffer (300 mM NaOH and 1 mM Na_2_EDTA, pH>13) and electrophoresed at 4°C for 50 min at 30 V and 300 mA. Then, slides were rinsed with sodium acetate (300 mM) and absolute ethanol (70%) solution (Carlo Erba) for 30 min, dehydrated (absolute ethanol for 2 h), re-hydrated (70% ethanol for 5 min) and stained, just before analysis, with 30 µM ethidium bromide (Bio-Rad). For each sample two slides were set up, and images of 1000 randomly selected nuclei (500 from each of the two replicate slides) were analysed by a computerized Image Analysis System (Delta Sistemi, Rome, Italy) fitted with a Leica DM BL fluorescence microscope at 250X magnification. The DNA migration pattern was evaluated by calculating the tail length (TL, in µm from the estimated leading edge of the head region to the leading edge of the tail), the percentage of migrated DNA (% DNA in the tail, calculated as the integrated intensity of DNA in the tail divided by the integrated intensity of DNA for the total image, multiplied by 100) and the tail moment (TM, the fraction of DNA in the tail multiplied by tail length), which are the generally reported comet parameters, although the % DNA in the tail is the most recommended one, due to the advantage that it gives some ‘feel’ for what the comet looks like [23]. Two hours treatment with 10 µM methyl methanesulfonate (MMS, Sigma), a well known DNA damaging agent that increases DNA migration, was included as a positive control. The 75^th^ percentile of comet data distribution was used to summarize the results on DNA migration pattern [24].

### 6. Statistical analysis

To evaluate cell viability, a comparison between exposed and sham-exposed cultures was performed by applying the two-tailed paired Student's *t*-test, with P values lower than 0.05 considered statistically significant.

The Kolmogorov-Smirnov (KS) test was employed to analyze flow cytometric measurements and comet assay data to compare exposed and sham-exposed samples. Statistical significance was set at P<0.01. In all cases MATLAB^®^ (Natik, MA, USA) software was employed.

## Results

### 1. Cell viability and growth

**Figure 1:**
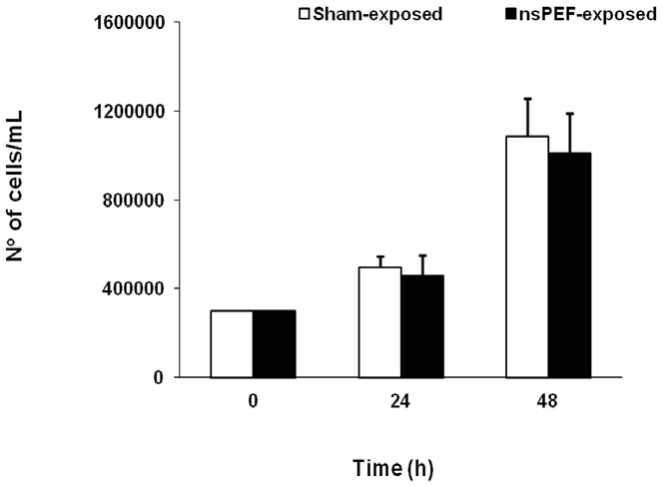
Cell growth remained unaffected after pulse exposure. Jurkat cell growth after a single, 60 ns, 2.5 MV/m pulse. Mean ± SD of 7 independent experiments.

A single pulse of 60 ns duration and 1.0, 1.5, or 2.5 MV/m amplitude does not affect the viability and growth of Jurkat cells. In all cases cell viability was at least 85% for both sham and exposed cells. In Fig. 1 the number of viable cells/mL is presented for 2.5 MV/m amplitude exposed and sham-exposed samples immediately post pulse (0 h) and after 24 and 48 h of growth. Data are presented as mean ± standard deviation (SD) of 7 independent experiments.

### 2. Plasma membrane integrity

**Figure 2:**
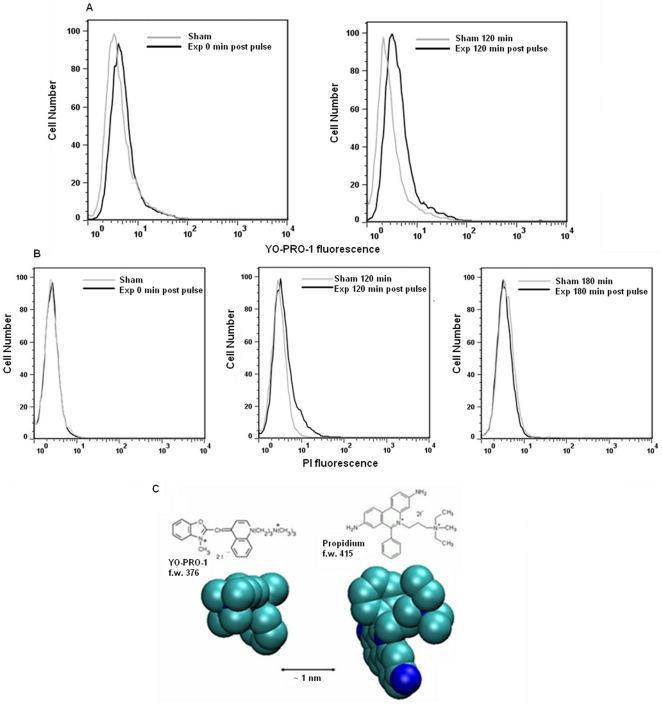
Jurkat cells plasma membrane was selectively permeabilized. Plasma membrane permeabilization of Jurkat cells after 1 pulse, 60 ns, 2.5 MV/m, presented as representative fluorescence histograms for A) YO-PRO-1 uptake immediately (0 min) and 120 min post pulse and B) PI uptake immediately, 120 min and 180 min post pulse. C) Molecular structures and 3-D models (van der Waal's radii) of the dyes.

A direct effect of a single pulse of 60 ns on plasma membrane integrity was detected by YO-PRO-1 staining; cells exposed in presence of the dye showed a pulse amplitude-dependent increase in YO-PRO-1 fluorescence with respect to sham exposed ones. A total of 9 independent experiments were carried out for each pulse amplitude, and an average increase of 28%, 36%, and 58% was recorded immediately after pulsing for 1.0, 1.5, and 2.5 MV/m, respectively. Time-course experiments with 2.5 MV/m pulses indicate that membrane permeability to YO-PRO-1 persists at least 120 min post pulse, as reported in Fig. 2A for a representative experiment. Cells remain initially impermeable to PI at this pulse dose, although a delayed PI uptake was observed at 60–120 min post pulse, similar to other reports [25] and it was recovered within 3 hours post pulse (Fig. 2B). A total of 10 independent experiments were carried out.

### 3. DNA migration pattern

**Figure 3:**
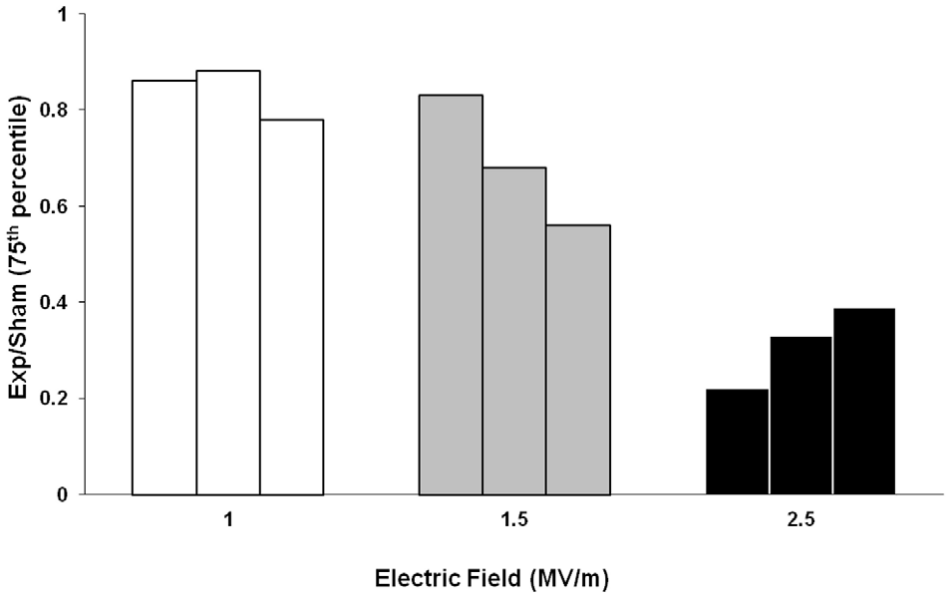
nsPEFs affect DNA migration in a dose-dependent fashion. Dose-response for Jurkat cell DNA migration after exposure to a single, 60 ns pulse. The 75^th^ percentile of the distribution of exposed cells normalized to sham-exposed ones is presented for each of three independent experiments carried out at 1.0, 1.5, and 2.5 MV/m. Data refer to % DNA in the tail.

A single pulse of 60 ns duration and 1.0, 1.5, and 2.5 MV/m amplitudes induces alterations of the DNA migration pattern of Jurkat cells in the alkaline comet assay. In particular, a statistically significant reduction of DNA migration was recorded in exposed cells with respect to sham-exposed ones, when analyzed immediately after the exposure. The results obtained from three independent experiments are summarized in Table 1, where the 75^th^ percentile of distribution of % DNA in the tail, tail length and tail moment are reported. Data refer to 1000 nuclei analyzed for each condition. The 75^th^ percentile value is always lower (P<0.01) in pulsed cells than in sham-exposed ones. The reduction in DNA migration is dose-dependent: the effect increases with increasing pulse amplitude. The dose-response relationship is shown for percentage DNA in the tail in Fig. 3, where the 75^th^ percentile of the distribution of exposed cells normalized to sham-exposed ones is presented for each of the 3 independent experiments carried out at each pulse amplitude. Note that the reduction of DNA migration pattern induced at the highest pulse amplitude tested is reversible. It is no longer evident at 60 min, as shown in Table 2.

**Table 1:** DNA migration pattern of Jurkat cells immediately after nsPEF exposure at 1.0, 1.5, 2.5 MV/m.

	% DNA in the tail		Tail length		Tail Moment	
Field strength	Sham	Exp	Sham	Exp	Sham	Exp
**1.0 MV/m**	14	12*	30	27*	5	4*
	17	15*	35	29*	7	5*
	23	18*	42	32*	9	7*
**1.5 MV/m**	23	19*	43	32*	9	7*
	19	13*	42	35*	8	5*
	16	9*	38	23*	6	3*
**2.5 MV/m**	9	2*	22	10*	3	1*
	9	3*	22	11*	3	1*
	46	18*	46	30*	16	6*

For each pulse amplitude, 3 independent experiments were carried out; each data point represents the 75^th^ percentile of the distributions. * P<0.01 (one sided Kolmogorov-Smirnov test; sham *vs* exposed samples).

**Table 2:** Time course of DNA migration pattern for 60 ns, 2.5 MV/m nsPEF exposed and sham-exposed Jurkat cells.

Experiment		% DNAin the tail		Taillength		TailMoment	
	Post pulse time (min)	Sham	Exp	Sham	Exp	Sham	Exp
1	**0**	17	13*	32	30*	7	5*
	**20**	16	10*	27	24*	5	3*
	**60**	17	16	29	28	4	4
2	**0**	13	7*	30	22*	5	3*
	**20**	8	6*	25	19*	3	2*
	**60**	11	13	40	40	6	6

Results are presented as 75^th^ percentile of the distributions.

*P<0.01 (one sided Kolmogorov-Smirnov test; sham *vs* exposed samples).

**Figure 4:**
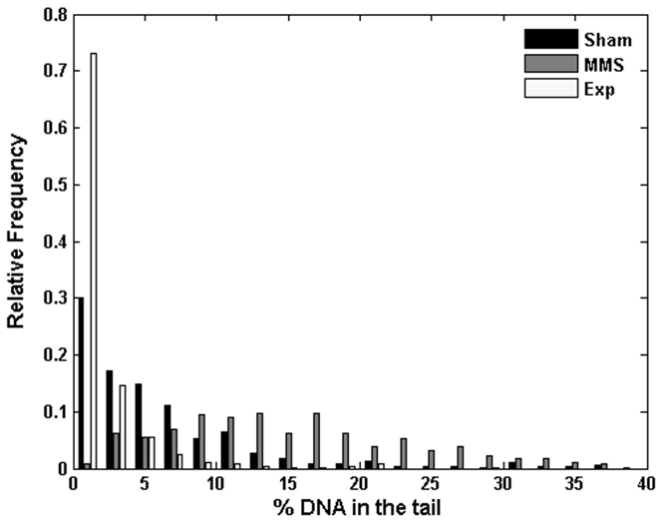
DNA migration pattern of Jurkat cells under nsPEFs *vs* MMS. Distribution of % DNA in the tail for Jurkat cells following nsPEF exposure (Exp), sham exposure (Sham) and 2 hr exposure to 10 µM of methyl methanesulfonate (MMS). Results refer to a representative experiment carried out at 1 pulse, 2.5 MV/m, 60 ns.

Interestingly, the observed effect is opposite to the one displayed by Jurkat cells treated for 2 h with 10 µM MMS, which causes single-strand breaks and enhancement of DNA migration in the comet assay [26]. The comparison among sham-exposed, 2.5 MV/m exposed, and methyl methanesulfonate-treated cells in terms of distribution of % DNA in the tail is shown in Fig. 4 for a representative experiment.

## Discussion

We have presented here evidence for nanosecond, megavolt-per-meter, pulsed electric field effects on the migration of DNA in the alkaline comet assay that are indicative of pulse-induced structural changes in DNA in the nucleus. A single electric pulse of 60 ns duration at 1.0, 1.5, and 2.5 MV/m amplitudes induces a statistically significant, dose-sensitive, and transient reduction of DNA migration in Jurkat cells, but does not affect cell viability. In spite of the extremely high power exhibited by nsPEFs, the observed effect can not be regarded as a thermal effect due to the low energy pulsing conditions [12]–[14].

In this study, the sensitivity of the alkaline comet assay has been improved by increasing the duration of electrophoresis so that a significant migration in the DNA of control cells is observed, and by analyzing 1000 nuclei rather than the 50–100 commonly considered to be sufficient [24]. In this way we can detect small but significant effects such as the reduction in DNA migration of nsPEF-exposed cells reported here.

The most likely explanation for our results is a pulse-induced, transient, conformational change in the living cell nucleoprotein. Although decreases in DNA migration in the comet assay are usually attributed to crosslinking (DNA-DNA or DNA-protein) [27], this mechanism is not the most likely for the reported observation, since the transient reduction of DNA migration induced by nsPEFs in our exposure conditions was detected in the absence of cytotoxic effects [28]. This may be a low-dose phenomenon, since when a stronger (6 MV/m) single 60 ns pulse was delivered to Jurkat and HL-60 cells, irreversible DNA damage (an increase in comet length) was detected, in association with a reduction of cell viability [29].

Results consistent with our hypothesis — the formation of DNA aggregates and conformational changes of the molecular structure after exposure to electric pulses — have been previously reported [30].

Chen and co-workers showed a strong nsPEFs effect on the HL-60 cell nucleus, under exposure conditions comparable to ours [7]. In particular, they observed after a single, 60 ns, 2.6 MV/m pulse a fluorescence quenching of acridine orange (AO), a DNA-intercalating dye, and they suggested that a nsPEF-induced change in DNA conformation was responsible for the reduction in AO fluorescence. Our results support this hypothesis, since the reduction in DNA migration that we observed can also be explained by the occurrence of a transient chromatin condensation in the pulse-exposed Jurkat cells. Furthermore, our observations extend those reported by Chen et al. regarding plasma membrane permeability. They claimed that only a delayed, secondary membrane permeabilization (PI uptake) occurred, but we have shown an immediate and long lasting YO-PRO-1 influx in addition to the delayed uptake of PI that recovered 2 to 3 hours post pulse. YO-PRO-1 presents a smaller cross-section than PI and is a more sensitive detector of plasma membrane poration (Fig. 2C), a property that has enabled the use of YO-PRO-1 as a sensitive indicator of early apoptosis [21]. Our results on plasma membrane electropermeabilization on the whole could be explained as a primary effect since YO-PRO-1 uptake was recorded right after the exposure, reflecting the immediate formation of nanopores. As a matter of fact, it has been demonstrated that the initial effect of nsPEFs is a sudden change of charge distributions along the membranes [31]. Then, nanopores may undergo a subsequent enlargement giving rise to a secondary, transient entry of PI, and reseal before reaching such a size sufficient to trigger membrane disruption [1]. As a matter of fact the observed nsPEFs induced membrane permeabilization to YO-PRO-1 and PI was not associated to cell death.

A correlation between the plasma membrane electropermeabilization and the effect on DNA migration is likely to exist due to the electrical continuity between the plasma membrane and the nuclear membranes. Nuclear DNA conformations will thus be influenced by the electric field arising from the surface potential at the nuclear membrane [32].

Overall, this work has provided a new demonstration of the possibilities for targeting and manipulating intracellular structures with intense nanosecond electric pulses, under conditions that do not affect cell viability but that still affect the integrity of the plasma membrane.

## References

[1] Joshi RP, Schoenbach KH (2010). Bioelectric effects of intense ultrashort pulses.. Critical Reviews in Biomedical Engineering.

[2] Vernier PT, Sun Y, Marcu L, Craft CM, Gundersen MA (2004). Nanoelectropulse-Induced Phosphatidylserine Translocation, Biophys. J..

[3] Vernier PT, Ziegler MJ, Sun Y, Gundersen MA, Tieleman P (2006a). Nanopore-facilitated, voltage-driven phosphatidylserine translocation in lipid bilayers—in cells and in silico.. Phys. Biol.

[4] Vernier PT, Sun Y, Gundersen MA (2006b). Nanoelectropulse-driven membrane perturbation and small molecule permeabilization.. BMC Cell Biol.

[5] Ibey BL, Mixon DG, Payne JA, Bowman A, Sickendick K (2010). Plasma membrane permeabilization by trains of ultrashort electric pulses.. Bioelectrochemistry.

[6] Schoenbach KH, Beebe SJ, Buescher ES (2001). Intracellular Effect of Ultrashort Electrical Pulses.. Bioelectromagnetics.

[7] Chen N, Schoenbach KH, Kolb JF, Swanson RJ, Garner AL (2004). Leukemic cell intracellular responses to nanosecond electric fields.. Biochem Biophys Res Commun.

[8] Buescher ES, Schoenbach KH (2003). Effects of Submicrosecond, High Intensity Pulsed Electric Fields on Living Cells - Intracellular Electromanipulation.. IEEE Trans Dielectr and Electr Ins.

[9] Tekle E, Oubrahim H, Dzekunov SM, Kolb JF, Schoenbach KH (2005). Selective field effects on intracellular vacuoles and vesicle membranes with nanosecond electric pulses.. Biophys J.

[10] Vernier PT, Sun Y, Marcu L, Salemi S, Craft CM (2003). Calcium bursts induced by nanosecond electric pulses.. Biochem Biophys Res Commun.

[11] Beebe SJ, Fox PM, Rec LJ, Somers K, Stark RH (2002). Nanosecond Pulsed Electric Field (nsPEF) Effects on Cells and Tissues: Apoptosis Induction and Tumor Growth Inhibition.. IEEE Trans Plasma Sci.

[12] Chen X, Kolb JF, Swanson RJ, Schoenbach KH, Beebe SJ (2010). Apoptosis initiation and angiogenesis inhibition: melanoma targets for nanosecond pulsed electric fields.. Pigment Cell Melanoma Res.

[13] Nuccitelli R, Pliquett U, Chen X, Ford W, Swanson RJ (2006). Nanosecond pulsed electric fields cause melanomas to self-destruct, Biochem Biophys Res Commun.

[14] Nuccitelli R, Chen X, Pakhomov AG, Baldwin WH, Sheikh S (2009). A new pulsed electric field therapy for melanoma disrupts the tumor's blood supply and causes complete remission without recurrence.. Int J Cancer.

[15] Pakhomov AG, Kolb JF, White JA, Joshi RP, Xiao S (2007). Long-Lasting Plasma Membrane Permeabilization in Mammalian Cells by Nanosecond Pulsed Electric Field (nsPEF).. Bioelectromagnetics.

[16] Pakhomov AG, Bowman AM, Ibey BL, Andre FM, Pakhomova ON (2009). Lipid nanopores can form a stable, ion channel-like conduction pathway in cell membrane.. Biochem Biophys Res Commun.

[17] Bowman AM, Nesin ON, Pakhomova ON, Pakhomov AG (2010). Analysis of plasma membrane integrity by fluorescent detection of Tl^+^ uptake.. J Membr Biol.

[18] Vernier PT, Sun Y, Chen MT, Gundersen MA, Craviso GL (2008). Nanosecond electric pulse-induced calcium entry into chromaffin cells.. Bioelectrochemistry.

[19] Palumbo R, Brescia F, Capasso D, Sannino A, Sarti M (2008). Exposure to 900 MHz radiofrequency radiation induces caspase-3 activation in proliferating human lymphocytes.. Radiat Res.

[20] Kolb JF, Kono S, Schoenbach KH (2006). Nanosecond pulsed electric field generators for the study of subcellular effects.. Bioelectromagnetics.

[21] Idziorek T, Estaquier J, De Bels F, Ameisen JC (1995). YOPRO-1 permits cytofluorometric analysis of programmed cell death (apoptosis) without interfering with cell viability.. J Immunol Methods.

[22] Singh NP, McCoy MT, Tice RR, Schneider EL (1988). A simple technique for quantitation of low level of DNA damage in individual cells.. Exp Cell Res.

[23] Zeni O, Scarfì MR (2010). DNA damage by carbon nanotubes using the single cell gel electrophoresis technique. In: Balasubramanian K, Burghard M editors. Carbon Nanotubes: Methods and Protocols-Methods in Molecular Biology, Vol. 625..

[24] Lovell DP, Omori T (2008). Statistical issues in the use of the comet assay.. Mutagenesis.

[25] Deng J, Schoenbach KH, Buescher ES, Hair PS, Fox PM (2003). The effects of intense submicrosecond electrical pulses on cells.. Biophys J.

[26] Collins AR, Dusinska M, Horska A (2001). Detection of alkylation damage in human lymphocytes DNA with the comet assay.. Acta Biochim Pol.

[27] Tice RR, Yager JW, Andrews P, Crecelius E (1997). Effect of hepatic methyl donor status on urinary secretion and DNA damage in B6C3F1 mice treated with sodium arsenite.. Mutat Res.

[28] Merk O, Speit G (1999). Detection of crosslinks with the Comet assay in relationship to genotoxicity and cytotoxicity.. Environ Mol Mutagen.

[29] Stacey M, Stickley M, Fox PM, Statler V, Schoenbach KH (2003). Differential effects in cells exposed to ultra-short high intensity electric fields: cell survival, DNA damage and cell cycle analysis.. Mutat Res.

[30] Porschke D, Meier HJ, Ronnenberg J (1984). Interaction of nucleic acid double helices induce by electric field pulses.. Biophys Chem.

[31] White JA, Pliquett U, Blackmore PF, Joshi RP, Schoenbach KH (2011). Plasma membrane charging of Jurkat cells by nanosecond pulsed electric fields.. Eur Biophys J.

[32] Matzke AJM, Matzke MA (1991). The electrical properties of the nuclear envelope, and their possible role in the regulation of eukaryotic gene expression.. Bioelectrochemistry and Bioenergetics.

